# Addenbrooke's Hospital, Cambridge

**Published:** 1900-02-24

**Authors:** 


### ADDENBBROOKE'S HOSPITAL, CAMBRIDGE.

THE following remarks by the *Cambridge Independent Press* may be of interest as they express a side of the Addenbrooke's question which we did not press: "I note that Sir Henry Burdett's paper, THE HOSPITAL, has an article upon Addenbrooke's, in which it draws attention to the fact that some of the reforms recently adopted by the Governors embody principles which he recommended, and which the Court had, presumably at the instigation of some 'obstructive old gentlemen,' previously rejected. It is due to Sir Henry Burdett to at once admit that this is so, but it was, perhaps wisely, not mentioned in the report of the committee. To the 'obstructive old gentlemen' Sir Henry's name had become as the red rag to the bull, simply because in their eyes 'Burdett' spelled 'Progress'"

